# Epidermal Growth Factor Receptor Mutation Enhances Expression of Cadherin-5 in Lung Cancer Cells

**DOI:** 10.1371/journal.pone.0158395

**Published:** 2016-06-30

**Authors:** Ming-Szu Hung, I-Chuan Chen, Jr-Hau Lung, Paul-Yann Lin, Ya-Chin Li, Ying-Huang Tsai

**Affiliations:** 1 Department of Thoracic Oncology, Division of Pulmonary and Critical Care Medicine, Chang Gung Memorial Hospital, Chiayi, Taiwan; 2 Department of Medicine, College of Medicine, Chang Gung University, Taoyuan, Taiwan; 3 Department of Respiratory Care, Chang Gung University of Science and Technology, Chiayi Campus, Chiayi, Taiwan; 4 Department of Emergency Medicine, Chang Gung Memorial Hospital, Chiayi, Taiwan; 5 Department of Medical Research, Chang Gung Memorial Hospital, Chiayi, Taiwan; 6 Department of Anatomic Pathology, Dalin Tzu Chi Hospital, Buddhist Tzu Chi Medical Foundation, Chiayi, Taiwan; 7 Department of Respiratory Care, College of Medicine, Chang Gung University, Taoyuan, Taiwan; University of Navarra, SPAIN

## Abstract

Epidermal growth factor receptor (EGFR) activation has been shown to play a critical role in tumor angiogenesis. In this study, we investigate the correlation between EGFR mutations and cadherin-5 (CDH5), which is an angiogenic factor, in lung cancer cells. Increased expression CDH5 is observed in lung cancer cells with EGFR mutations. Stable lung cancer cell lines expressing mutant (exon 19 deletion E746-A750, and exon 21 missense mutation L858R) and wild type EGFR genes are established. A significantly higher expression of CDH5 is observed in exon 19 deletion stable lung cancer cells and mouse xenografts. Further studies show that expression of CDH5 is decreased after the inhibition of EGFR and downstream Akt pathways in lung cancer cells with EGFR mutation. In addition, mutant EGFR genes potentiates angiogenesis in lung cancer cells, which is inhibited by CDH5 siRNA, and potentiates migration and invasion in lung cancer cells. Our study shows that mutant EGFR genes are associated with overexpression of CDH5 through increased phosphorylation of EGFR and downstream Akt pathways. Our result may provide an insight into the association of mutant EGFR and CDH5 expression in lung cancer and aid further development of target therapy for NSCLC in the future.

## Introduction

The epidermal growth factor receptor (EGFR) pathway plays an important role in the growth, proliferation, and survival of many solid tumors, including non-small cell lung cancer (NSCLC) [1]. As a result, it is an attractive target for target therapy. A subgroup of patients with NSCLC having specific mutations in the tyrosine kinase domain of EGFR gene, which correlates with favorable clinical responsiveness to EGFR tyrosine kinase inhibitors (EGFR-TKI) such as gefitinib, erlotinib, and afatinib therapy, has been noted [2–4]. All mutations appear to be limited to exons 18, 19, 20, and 21 of the EGFR gene [5]. Missense mutations in exon 21 (L858R) and in-frame deletions within exon 19 (delE746-A750) have been shown to be the most frequent EGFR-TKI sensitive mutations (80%) in NSCLC [6, 7].

EGFR activation is related to the stimulation of tumor angiogenesis, which is essential to growth, proliferation, and metastasis of cancer cells [1]. Expression of EGFR has also been reported to be associated with the expression of angiogenic factors, such as TGF-α and [8] VEGF in human cancers [9]. In addition, EGFR mutation has been reported to be related to an increased expression of IL-6 [10] and VEGF [11] in NSCLC cells and tissues. Cadherin-5, also known as VE-cadherin, CDH5, and CD144, is a membrane protein and is encoded by the human gene *CDH5*. CDH5 is localized at intercellular junctions of endothelial cells and plays an important role in the control of vascular integrity and permeability, and contributes to endothelial cell assembly in tubular structure [12]. CDH5 is indispensable to the survival signal of VEGF on endothelial cells [13]. The expression of VEGF and CDH5 in circulation microvesicles has been reported to be associated with distant metastasis in lung cancer [14]. However, the association of EGFR mutations with expression of CDH5 has not been reported in the literature.

In this study, we studied the association of CDH5 expression with common EGFR mutations (exon 19 delE746-A750 and exon 21 L858R) in lung cancer cells and mouse xenograft models. Stable lung cancer cells transfected with wild type and mutant EGFR genes were also established. The mechanism about how CDH5 was regulated by mutant EGFR genes and the association of EGFR mutation with angiogenesis, migration, and invasion of lung cancer cells were further studied.

## Material and Methods

### Cell culture

The NSCLC cell lines A549 (ATCC CCL-185), H460 (ATCC HTB-177), H322 (ATCC CRL-5883), H838 (ATCC CRL-5844), H1703 (ATCC CRL-5889), H2228 (ATCC CRL-5935), and H1975 (ATCC CRL-5908) were purchased from American Type Culture Collection (Manassas, Virginia, United States). PC9 was a generous gift from Professor Pan-Chyr Yang at National Taiwan University, Taipei, Taiwan. PC9 and H1975 cell lines have EGFR mutations (delE746-A750 for PC9, L858R and T790M for H1975) [15]. Human umbilical vein endothelial cell line, HUVEC (H-UV001), was purchased from Bioresource Collection and Research Center (Hsinchu City, Taiwan). Cells were grown in RPMI-1640 complete growth medium supplemented with 10% fetal calf serum, 30 ng/ml EGF, 10 units/ml penicillin, and 10 μg/ml streptomycin at 37°C and 5% CO2. HUVEC cells were grown in 90% Medium 199 with 25 U/ml heparin, 30 ug/ml endothelial cell growth supplement (ECGS) adjusted to contain 1.5 g/L sodium bicarbonate, 10 units/ml penicillin and 10 μg/ml streptomycin, and 10% fetal calf serum. For inhibition to the phosphorylation of EGFR and Akt, PC9 and H1975 lung cancer cells were treated with indicated concentrations of EGFR-TKI (Afatinib, LC Laboratories, Woburn, MA) and Akt (MK-2206, Selleckchem, Houston, TX) inhibitors at indicated durations.

### RNA extraction, cDNA synthesis, and real-time polymerase chain reaction (PCR)

RNA was extracted by using RNeasy Mini Kit (QIAGEN, Hilden, Germany) from cell pellets according to the manufacturers’ instructions. Total RNA was then transcripted to cDNA using iScript^™^ cDNA Synthesis Kit (Bio-Rad Laboratories, Munich, Germany). Next, 2 μl of reverse-transcribed cDNA was subjected to real time PCR using the iQ^™^ SYBR^®^ Green supermix with a total volume of 20 μl and the Bio-Rad CFX96^™^ quantitative PCR system (Bio-Rad Laboratories, Munich, Germany). The following primers were used for PCR: CDH5, CCTACCAGCCCAAAGTGTGT (sense) and GACTTGGCATCCCATTGTCT (antisense); β-Actin, CCTGGACTTCGAGCAAGA GATG (sense) and AGGAAGGAAGGCTGGAAGAGTG (antisense). A typical protocol included a 95°C denaturation step for 3 minutes followed by 35 cycles with a 95°C denaturation for 20 seconds, 60°C annealing, and extension for 30 seconds. Detection of the fluorescent product was carried out at the extension step. Melting curve detection and analysis were performed by additional 80 cycles with a 55°C denaturation with a 0.5°C increase after each cycle. Finally, the real-time PCR products were held at 4°C. Relative CDH5 expression was analyzed by the 2(-Delta Delta Ct) method using β-Actin as the internal control [16].

### Establishment of lung cancer stable cell lines expressing wild type and mutant EGFR genes

A retroviral system was used for transfection of EGFR genes into A549 lung cancer cells. In brief, pBabe-puro vectors (Addgene, Cambridge, MA) containing the cDNA of wild type EGFR and mutant EGFRs (delE746-A750 in exon 19, and L858R in exon 21) were transfected into HEK 293 Phoenix ampho packaging cells (ATCC, Manassas, VA) using Fu-GENE6 transfection reagent (Roche, Lewes, UK). The supernatant was collected for transduction of retrovirus into A549 lung cancer cells 48 hours after transfection. After being selected with puromycin for 3 weeks, the remaining cell colonies were amplified and checked for EGFR expression and used for further analysis.

### Protein extraction and western blot analysis

Whole protein was extracted and added with phosphatase inhibitor and protease inhibitor. Proteins were separated on 8% sodium dodecyl sulfate (SDS)–polyacrylamide gels and transferred to Immobilon-P membranes (Millipore, Billerica, MA). The following primary antibodies, EGFR (Santa Cruz Biotechnology, Dallas, TX), phospho-EGFR (Tyr1068, Tyr 1173 and Tyr 845), phospho-Stat3 (Tyr 705), Stat3, phospho-Erk1/2 (Thr202/Tyr204), Erk1/2, HER2, (Cell Signaling, Beverly, MA), Akt (Santa Cruz Biotechnology), phospho-Akt (Ser473) (Santa Cruz Biotechnology), CDH5 (Santa Cruz Biotechnology), and β-actin (Santa Cruz Biotechnology), were used. After primary antibody and antigen complexes were bound to specific secondary antibodies, an enhanced chemiluminescence (ECL) blotting analysis system (GE Healthcare Life Sciences, Piscataway, NJ) was used for antigen-antibody detection. Densitometry of western blot was calculated by using ImageJ (v1.44m for Windows, National Institutes of Health).

### Transwell co-culture assay

HUVEC cells (3x10^4^) were cultured in 35-mm 6 well dual-layered culture dishes. After 24 hours, wild type and mutant EGFRs transfected cells (5x10^4^) were seeded onto the cell culture insert with 0.4-μm micropores on the bottom (Becton Dickinson, Franklin Lakes, NJ, USA) and placed in the wells growing HUVEC cells. HUVEC cells were collected on day 5 after co-culturing, and viable cells were then counted with a hemocytometer.

### Transfection of siRNA

Pre-designed and validated CDH5 and universal negative control siRNA (Santa Cruz Biotechnology, Inc.) were used for transfection study. Transfection was performed using Lipofectamine^™^ RNAiMAX transfection reagent (Invitrogen Life Technologies, Carlsbad, CA, USA) according to the manufacturer's manual. The cells were plated in 6 well plates in antibiotic free media, and transfection was performed with cells at 80% confluency with a final concentration of 50 nM for each siRNA. At 72 hours after transfection, the cells were used for further studies.

### Immunofluorescence

For immunofluorescence microscopy, the cells were grown on coverslips, fixed in cold methanol for 10 minutes at—20°C, and blocked with 2% bovine serum albumin for 30 min. The cells were incubated with the primary anti—CDH5 (Santa Cruz Biotechnology) antibody in 2% bovine serum albumin for 1 hour at room temperature. The cells were washed with PBS and subsequently incubated with FITC—conjugated secondary antibodies in 2% bovine serum albumin for 1 hour at room temperature. After being washed with PBS, the cells were counterstained with DAPI and mounted in Vectashield (Vector Laboratories, Burlingame, CA). Images were acquired using a TCS SP5 confocal microscope (Leica, Wetzlar, Germany). The intensity of CDH5 was quantified by MetaMorph^®^ Microscopy Automation & Image Analysis Software (Molecular Devices, Sunnyvale, CA).

### Immunohistochemistry (IHC)

Formalin-fixed, paraffin-embedded tissues were cut into 4-μm sections, mounted on slides, deparaffinized with xylene, and dehydrated using a gradient ethanol series. Stable lung cancer cells were cultured in Nunc Lab-Tek^™^ II—Chamber Slide System and then fixed with formalin for further IHC study. Antigen retrieval was performed with citric acid (pH 6.0) at 97°C for 30 minutes, and followed by treatment with 3% hydrogen peroxide. The slides were incubated overnight at 4°C with antibodies against CDH5 and then incubated with specific secondary antibodies conjugated to horseradish peroxidase-linked labeled polymers. 3, 3’-Diaminobenzidine-positive substrate-chromogen was added to sections and incubated for 5 minutes for color development. Finally, the slides were counterstained with Harris's haematoxylin for 5 seconds, dehydrated, and mounted. The intensity of IHC staining was calculated by using ImageJ (v1.44m for Windows, National Institutes of Health).

### Xenografting

The animal study was approved by the Institutional Animal Care and Use Committee (IACUC) in Chang Gung Memorial Hospital, Chiayi branch. Six weeks old female BALB/c nude mice were purchased from BioLASCO Taiwan Co., Ltd (Taipei, Taiwan). Mice were bred at laboratory animal center in Chang Gung Memorial Hospital, Chiayi branch under the guidance of the Association for the Assessment and Accreditation of Laboratory Animal Care (AAALAC). After the stable cell lines were established, cells from one 150-cm^2^ flask was trypsinized and washed with PBS by centrifugation. Cell pellet that contains 1x10^6^ cells was suspended in PBS and injected to the flank areas of nude mice subcutaneously. BD Matrigel Matrix (BD Biosciences, Bedford, MA), a solubilized tissue basement membrane extract, was used to facilitate tumor formation in nude mice. Four mice were included in each groups of stable cell lines with a total of sixteen mice. The volume of the tumor on the animal after xenografting was assessed using a caliper and the tumor volume was calculated by the formula (L x W^2^/2). The health status of the mice were monitored every day. The mice would be euthanized if the average tumor diameter greater than 20 mm, ulceration, infection, or gangrene changes in the surface of tumor, poor intake, respiratory distress or abdominal distension, or body weight loss more than 25% developed. No mice died in the experimental period. Tumor volume was assessed twice a week for 6 weeks. All mice were sacrificed using CO2 euthanasia at the end of the study and tumors were excised for further studies.

### Cell Migration Assay

For wound-closure experiments, cells were plated in 10-cm plates and cultured to confluence. Cells were scraped with a p20 tip and transferred to pre-warmed fresh media. The healing of the gap was observed at indicated time points.

### Cell Invasion Assay

The invasion assays were performed in 24-well 6.5-mm diameter inserts (Corning, 8.0-mm pore size) coated with an indicator layer of growth factor reduced Matrigel (BD Biosciences, Bedford, MA). The cells were plated in the upper well in 0.2% serum and incubated with 5% FBS and 100 ng/ml fibronectin in the lower chambers. After 24 hours, cells in the upper chamber were removed with a cotton swab. Cells that have migrated into the lower chamber were fixed in 4% PFA and stained with 0.5% Crystal Violet. Filters were photographed and the total number of cells was then quantified.

### Statistical analysis

The student’s *t*-test was used to compare variables in different groups of samples unless otherwise specified. Statistical analysis was carried out by using GraphPad Prism 5 (GraphPad Software, Inc., La Jolla, CA). Data were expressed as mean ± SD. Significance was defined as *p* < 0.05 with two-sided analysis.

## Results

### EGFR mutations and CDH5 expression in lung cancer cells

**Fig 1:**
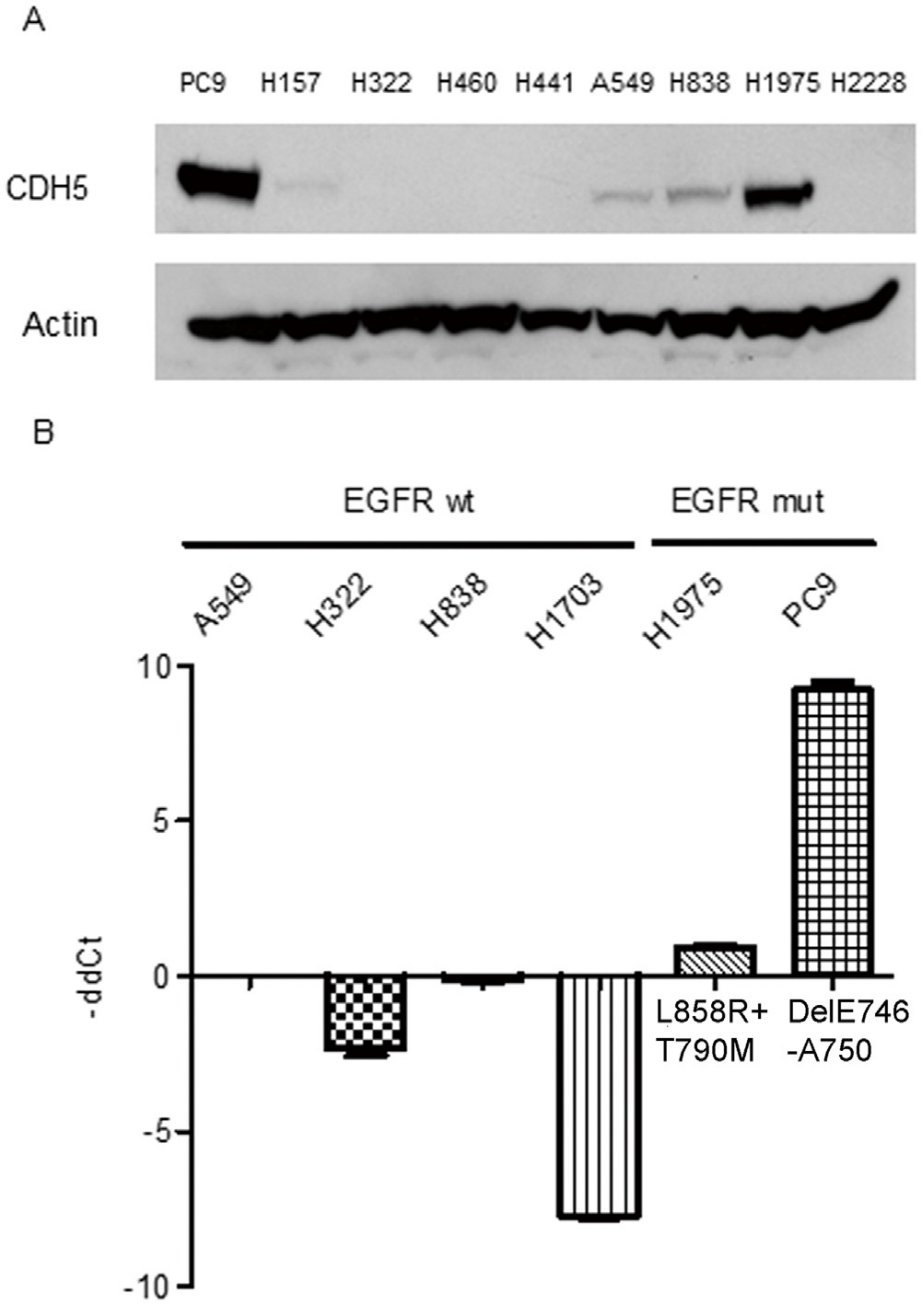
A. Western blot analysis of CDH5 in lung cancer cell lines. β-Actin was used as internal control. B. RT-PCR for the CDH5 gene in lung cancer cell lines. wt: wild type, mut: mutation. β-Actin gene was used as internal control. The expression of CDH5 gene was normalized to A549 lung cancer cells. Data shown are the mean ± SD of three independent experiments.

The association of EGFR mutations and CDH5 expression was studied in lung cancer cells using western blot analysis. An increased expression of CDH5 proteins was observed in PC9 and H1975 lung cancer cells with EGFR mutations (Fig 1A). Further RT-PCR analysis also showed an increased expression of CDH5 mRNA in lung cancer cells with EGFR mutations (Fig 1B).

### Establishment of lung cancer stable cells expressing wild type and mutant EGFR genes

**Fig 2:**
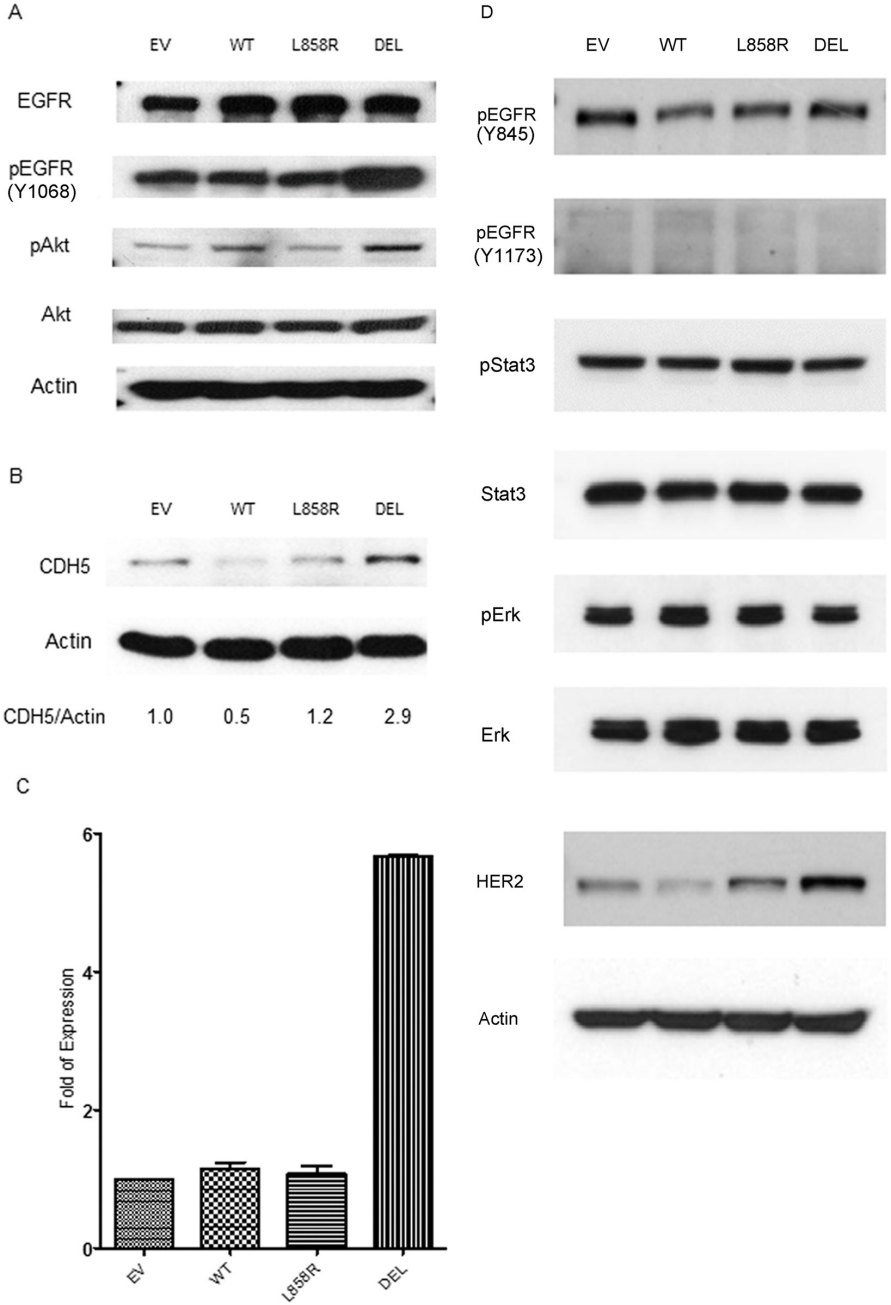
A. Western blot analysis for EGFR, pEGFR (Y1068), Akt, pAkt and B. CDH5 in A549 lung cancer cell line. A549 lung cancer cells were stably transfected with empty vector (EV), wild type (WT) and mutant (L858R, DEL) EGFR genes. Density of CDH5 was quantified and normalized to β-Actin using empty vector transfected cell lines as normal controls. C. RT-PCR for the CDH5 gene in A549 lung cancer cells transfected with empty vector (EV), wild type (WT) and mutant (L858R, DEL) EGFR genes. L858R: exon 21 misense mutation. DEL: exon 19 E746-A750 deletion mutation. Data shown are the mean ± SD of three independent experiments. D. Western blot analysis for EGFR, pEGFR (Y1068 and Y1173), pStat3, Stat3, pErk, Erk and HER2. β-Actin was used as the internal control. Y1068: Tyr1068; Y1173: Tyr 1173; Y845: Tyr 845.

To further validate the association of mutant EGFR and expression of CDH5 in an isogenic background, stable lung cancer cell lines expressing wild type and mutant EGFR genes were then established. A549 lung cancer cells were retrovirally transfected with vectors containing wild type and mutant EGFR genes (exon 19 E746-A750 deletion and exon 21 L858R missense mutations) as described in the material and method section. After selection with puromycin, resistant cell colonies were amplified. EGFR expression in stable cells were detected by western blot analysis (Fig 2A). Compared to wild type EGFR gene transfected cells, increased expressions of phospho-EGFR (Y1068) and phospho-Akt proteins were detected in exon 19 E746-A750 deletion mutation EGFR gene transfected A549 lung cancer stable cells (Fig 2A). No obvious difference in the expression of other phospho-EGFR (Y845 and Y1173), phospho-Stat3, and phospho-Erk was observed. Increased expression of HER2 was observed in exon 21 L858R and exon 19 E746-A750 deletion mutation EGFR genes transfected A549 lung cancer stable cells (Fig 2D). Increased expression of HER2 was observed in both EGFR exon 21 L858R missense and exon 19 E746-A750 deletion mutations lung cancer stable cells (Fig 2D).

### EGFR mutations and expression of CDH5 in lung cancer stable cells

The expressions of CDH5 mRNA and protein were detected in mutant and wild type EGFR genes transfected stable lung cancer cells. Higher expressions of CDH5 protein and mRNA in lung cancer stable cells transfected with exon 19 deletion EGFR gene (Fig 2B and 2C) compared to wild type EGFR gene transfected stable cells were observed.

**Fig 3:**
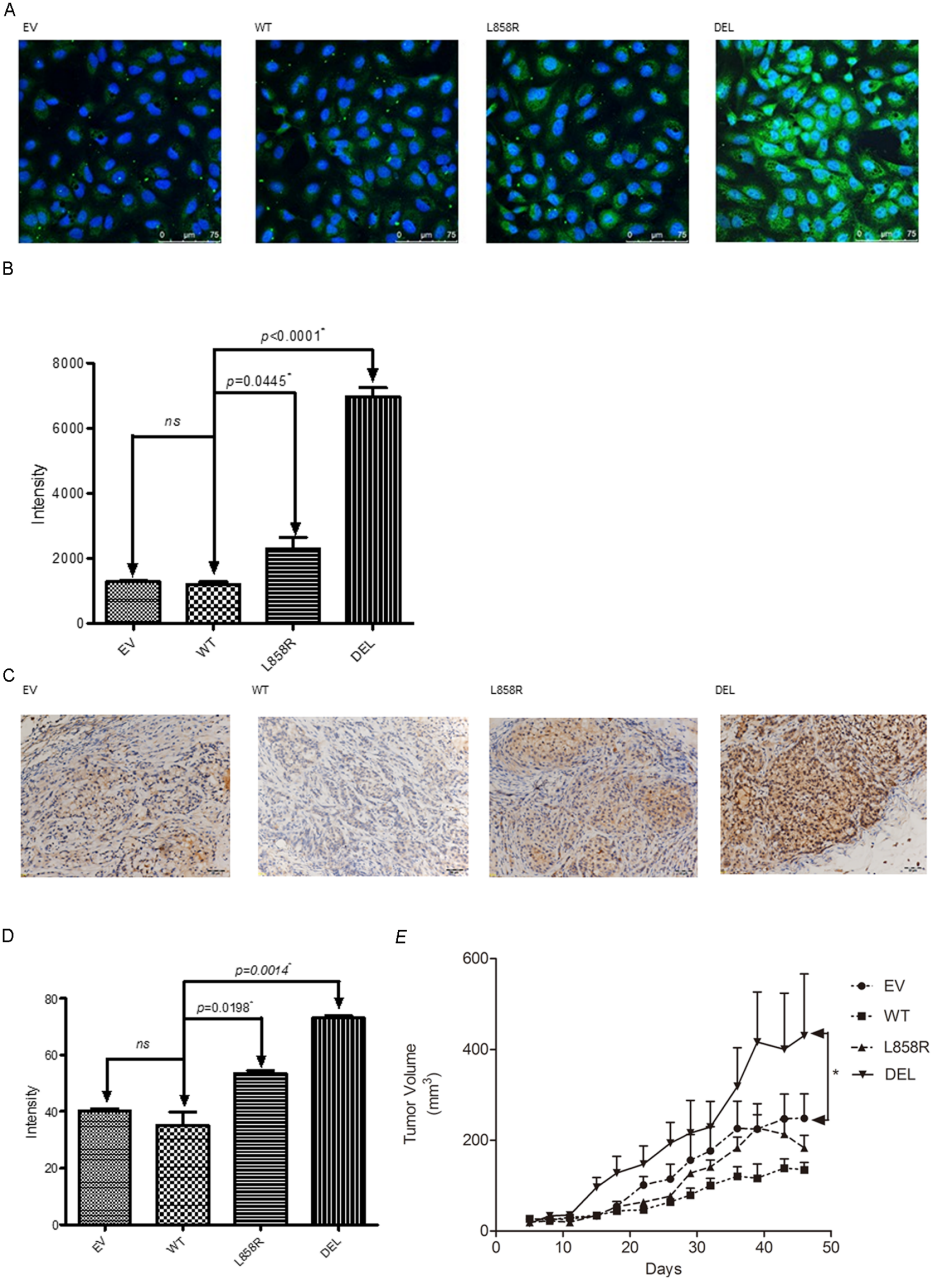
A. Immunofluorescent analysis of CDH5 in A549 lung cancer cells transfected with empty vector (EV), wild type (WT) and mutant (L858R, DEL) EGFR genes. CDH5 was stained as green color and nuclei were stained as blue color. Scale bar 75 μm. B. The intensity of TGFBI in each cells was quantified and shown as the mean ± SD of three independent experiments. * denotes *p*<0.05. C. CDH5 was determined by immunohistochemical (IHC) staining in mutant and wild type A549 lung cancer mouse xenografts. D. The expression of CDH5 was quantified and values of intensity are expressed as the mean ± SD of three independent experiments. * denotes *p*<0.05. E. A549 lung cancer cells nude mice xenograft model. Data points represent the average of tumor volume± SEM (*n* = 4 in each group). A549 lung cancer cells were transfected with empty vector (EV), wild type (WT) and mutant (L858R, DEL) EGFR genes. * denotes that tumor volume between EV and DEL group was statistically significantly different at *p* < 0.05 by two-way ANOVA.

Immunofluorescent study was used to study the expression of CDH5 protein in lung cancer stable cells. Overexpression of CDH5 was observed in both exon 21 L858R and exon 19 deletion mutant EGFR genes transfected stable cells compared to wild type EGFR gene transfected stable cells (Fig 3A). The expression of CDH5 protein was observed mainly in the cytoplasm and surface of cells. A significantly higher expression of CDH5 protein was observed in exon 19 deletion mutant EGFR gene transfected stable cells (Fig 3B).

To further validate our *in vitro* study results, a mouse xenograft model was established to study the correlation of expression of CDH5 and EGFR mutations *in vivo*. The expression of CDH5 was evaluated in tumors of A549 lung cancer stable cells using IHC study (Fig 3C). A significantly higher expression of CDH5 protein was observed in tumors with exon 19 deletion mutation (Fig 3D). Increased growth of tumor was also observed in tumors with exon 19 deletion mutation in the mouse xenograft model (Fig 3E).

### Correlation of CDH5 expression with EGFR and downstream Akt pathways

**Fig 4:**
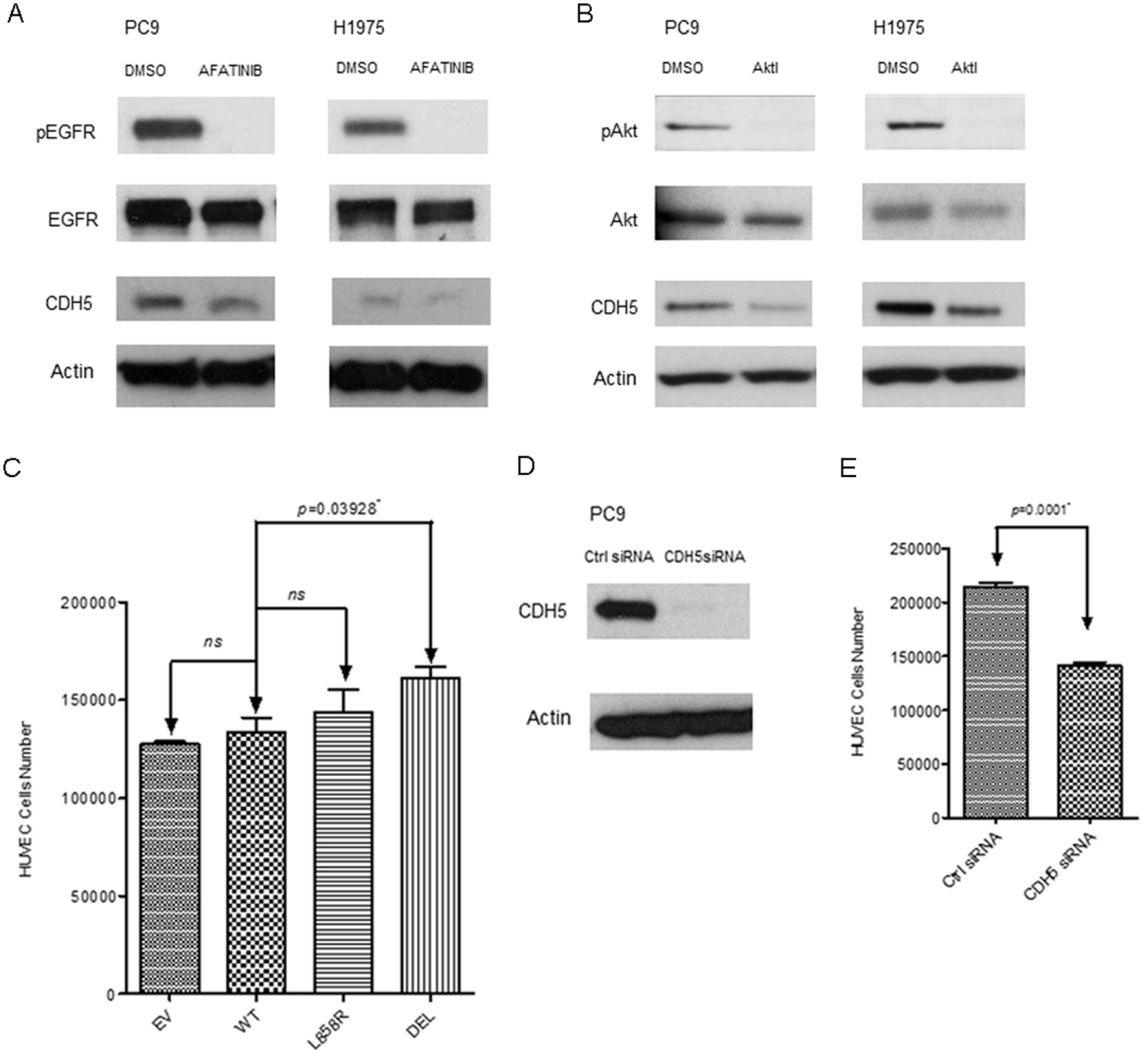
A. Western blot analysis of EGFR, pEGFR, and CDH5 in PC9 and H1975 lung cancer cells treated with indicated concentrations of Afatinib (0.001 μM for PC9 and 1 μM for H1975) for 72 hours. B. Western blot analysis of Akt, pAkt, and CDH5 in PC9 and H1975 lung cancer cells treated with 5 μM of MK-2206 Akt inhibitor (Akti). DMSO treated group was used as the control group. β-Actin was used as internal control. C. HUVEC cells were co-cultured with EGFR transfected A549 stable lung cancer cell lines in a trans-well system for 72 hours. Cells in each group were counted. D. Western blot analysis of CDH5 in PC9 lung cancer cells treated with control siRNA and CDH5 siRNA. E. HUVEC cells were co-cultured with control or CDH5 siRNA transfected A549 stable lung cancer cells in a trans-well system for 72 hours. Data shown are the mean ± SD of three independent experiments. * denotes *p*<0.05.

Inhibition of EGFR and downstream Akt pathways was performed to elucidate the association of CDH5 expression with EGFR and downstream Akt pathways. Afatinib, an EGFR-TKI inhibitor, was used to inhibit the phosphorylation of EGFR in PC9 and H1975 lung cancer cells (Fig 4A). After inhibition of the phosphorylation of EGFR, downregulation of CDH5 protein was observed in the lung cancer cells studied (Fig 4A).

The phosphorylation of Akt pathway was further inhibited by MK-2206, an Akt inhibitor (Fig 4B). After inhibition of the phosphorylation of Akt, downregulation of CDH5 protein was also observed in the lung cancer cells studied (Fig 4B). Our results showed that the expression of CDH5 was regulated by the phosphorylation of EGFR and downstream Akt pathways.

### EGFR mutations, CDH5, and angiogenesis in lung cancer cells

A trans-well co-culture system was then used to evaluate the angiogenic ability of mutant EGFR genes in promoting HUVEC cell growth. HUVEC cells were co-cultured with EGFR transfected A549 stable lung cancer cells in a trans-well system for 3 days. Significantly increased HUVEC cells were observed in the group co-cultured with A549 stable lung cancer cells transfected with exon 19 deletion mutant EGFR gene (Fig 4C).

CDH5 siRNA was then used to knock down the expression of CDH5 in PC9 lung cancer cells which has exon 19 deletion mutation (Fig 4D). Compared to control siRNA treated group, significantly decreased HUVEC cells were observed in the CDH5 siRNA treated group (Fig 4E). Our results showed that exon 19 deletion mutation was related to increased angiogenic ability in lung cancer cells, which was at least partially related to increased expression of CDH5.

### EGFR mutations, migration, and invasion in lung cancer cells

**Fig 5:**
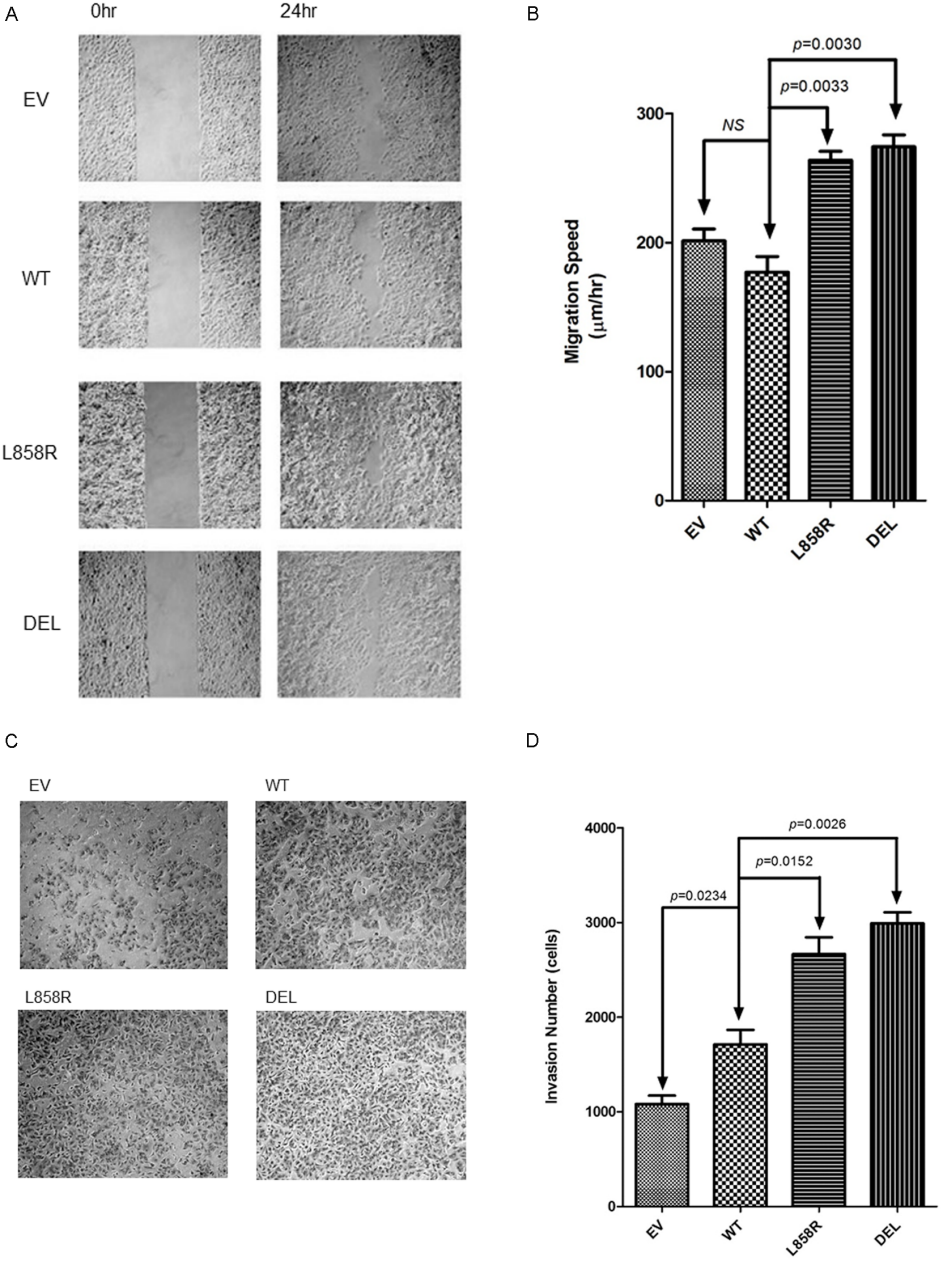
A. Cell migration assay of A549 lung cancer cells transfected with empty vector (EV), wild type (WT) and mutant (L858R, DEL) EGFR genes. B. Migration speed of A549 lung cancer stable cells. Data shown are the mean ± SD of three independent experiments. * denotes *p*<0.05. C. Cell invasion assay of A549 lung cancer cells transfected with empty vector (EV), wild type (WT) and mutant (L858R, DEL) EGFR genes. D. Invasion assay of A549 lung cancer stable cells. Data shown are the mean ± SD of three independent experiments.

CDH5, which is upregulated in exon 19 deletion mutant EGFR gene overexpressed lung cancer cells, is related to the ability of metastasis in lung cancer cells. We further studied the association of migration and invasion with EGFR mutant genes in lung cancer cells. Significantly increased migration (Fig 5A and 5B) and invasion (Fig 5C and 5D) were observed in both exon 19 deletion and exon 21 L858R missense mutant EGFR genes transfected lung cancer cells. In addition, higher migration and invasion abilities were observed in the exon 19 deletion mutant EGFR gene group.

## Discussion

In our study, we also observed that the increased expression of CDH5 was associated with EGFR mutations in lung cancer cells. Further study also showed that upregulation of CDH5 was associated with exon 19 deletion mutation of the EGFR gene *in vitro* and *in vivo*. The expression of CDH5 was regulated by phosphorylation and EGFR and downstream Akt pathways. In addition, exon 19 deletion mutation of the EGFR gene was associated with increased angiogenic ability in lung cancer cells, which was inhibited by a knockdown of CDH5 siRNA. To our knowledge, the association of CDH5 and EGFR mutations has not been reported in the literature. Increased growth of tumors with exon 19 deletion was also observed in mouse xenografts. Furthermore, we also showed that common EGFR mutations (exon 19 deletion and exon 21 missense L858R) were associated with increased migration and invasion in the lung cancer cells studied.

CDH5 is also important for tumor angiogenesis, and its expression is upregulated in the vasculature of breast carcinoma and is also identified as a metastasis marker in breast cancer [17–19]. CDH5 is also overexpressed in brain gliomas, correlates with tumor grades, and is an independent adverse prognostic predictor for glioblastoma multiforme patients [20]. In NSCLC tissues, the expression of CDH5 has been reported to be associated with lymph node metastasis and poor prognosis [21]. In our study, a higher expression of CDH5 was also observed in PC9 lung cancer cells with exon 19 deletion mutation and lung cancer cells transfected with exon 19 deletion mutant EGFR gene. Exon 19 deletion of the EGFR gene was observed to promote proliferation of HUVEC cells, which presented angiogenic ability. Knockdown of CDH5 was noted to inhibit proliferation of HUVEC cells. We thus propose that mutant EGFR genes may potentiate the potential of angiogenesis and metastasis through upregulation of CDH5. Our results are supported by the recent finding that exon 19 deletion mutation is associated with brain metastasis in lung adenocarcinoma patients [22].

Mutant EGFR genes have been reported to upregulate IL-6 and then activate the gp130/JAK/STAT3 pathway in primary human lung adenocarcinomas [10]. Inhibition of EGFR activation resulted in decreased expression of IL-8 in pancreatic cancer cells [23]. Our unpublished data also showed that overexpression of mutant EGFR genes were associated with upregulation of VEGF in lung cancer cells. Together with previous studies, our study further addresses the critical role of mutant EGFR genes in the promotion of angiogenesis in lung cancer cells. Increased migration and invasion abilities were also noted in the mutant EGFR genes transfected lung cancer cells in our study. Both exon 19 deletion and exon 21 missense L858R mutations are associated with increased cellular migration and invasion, and exon 19 deletion mutation shows a stronger effect in promoting migration and invasion. CDH5 has been reported to be associated with breast cancer metastasis [24] and aggressive melanoma [25]. Together with our findings, CDH5 may also contribute to an increased lung cancer metastasis. Since exon 21 missense mutation is also related to metastasis in lung cancer cells, other metastasis factors may be involved in mutant EGFR gene-related lung cancer metastasis. Further study is still warranted to further elucidate the association of metastasis factors and mutant EGFR genes.

Significantly upregulated CDH5 was observed in lung cancer cells which were transfected with exon 19 deletion mutation in our study. Both exon 19 deletion and exon 21 missense mutations, which are common EGFR mutations, have been proved to be associated with a favorable response to EGFR-TKIs such as gefitinib, erlotinib, and afatinib [26–28]. However, exon19 deletion mutation has been reported to be associated with a better outcome after EGFR-TKIs therapy than exon21 L858R mutation [27], which implies that exon 19 deletion mutation of the EGFR gene may have more distinct features than exon 21 L858R mutation. Increased EGFR phosphorylation was observed in stable lung cancer cells with exon 19 deletion mutation in our study. EGFR mutation has been reported to cause repositioning of critical residues surrounding the ATP-binding cleft of the tyrosine kinase domain of the EGFR receptor, and stabilize the interactions with ATP and EGFR-TKIs, leading to increased tyrosine kinase activity and drug sensitivity[29]. As a result, increased phosphorylation of EGFR in stable lung cancer cells with exon 19 deletion mutation may be due to distinct conformational changes within the catalytic pocket. In our study, increased phosphorylation of EGFR Y1068 was observed in exon 19 deletion stable lung cancer cells, which is similar to previous reports in lung cancer cells[30]. Increased phosphorylation of EGFR Y1068 activated downstream Akt pathway, and increased phosphorylation of Akt. Further inhibition of the Akt pathway showed decreased expression of CDH5 in EGFR mutant lung cancer cells. As a result, we postulated that mutant EGFR increased expression of CDH5 at least partially through increased phosphorylation of EGFR, Akt and downstream pathways. The Akt pathway also regulates cell proliferation and survival[31], and increased tumor growth was observed in tumors with mutant EGFR in our and other studies[32].

We observed that exon 19 deletion mutation was associated with upregulation of CDH5 *in vitro* and *in vivo* and higher angiogenic ability in the lung cancer cells studied, and the results support the finding that exon 19 deletion and exon 21 L858R mutation have distinct features. We proposed that higher angiogenic ability observed in lung cancer cells with exon 19 deletion was through increased phosphorylation of EGFR and downstream pathways, thus the growth of lung tumors with exon 19 deletion may rely more on angiogenesis. After EGFR-TKIs treatment, tumors with exon 19 deletion may be more susceptible to oncogenic shock induced by EGFR-TKIs[33]. Since CDH5 has been reported to be a potential target for cancer therapy [34], our results may also contribute to the future development of anti-CDH5 therapy in conjunction with EGFR-TKI therapy in lung cancer patients.

HER2 (also known as ErbB2) belongs to the same HER or ErbB family as EGFR (HER1/ErbBB1). After stimulated by various ligands including EGFR, TGF-α and others, EGFR forms homodimer with EGFR or heterodimer with HER2 (ErbB2)[35]. Increased expression of HER2 in lung cancer stable cells with mutant EGFRs, notably exon 19 deletion, was observed in our study. The heterodimer of EGFR with HER2 has been reported to induce a more potent phosphorylation and activation of EGFR than does EGFR homodimer[36], which may contribute to increase in phosphorylation of EGFR and downstream Akt pathways in our study. Although the mechanism by which mutant EGFR upregulated the expression of HER2 remains unknown, and further study is still needed.

In summary, our study shows that exon 19 deletion mutant EGFR gene is associated with an increased expression of CDH5 in lung cancer cells through increased phosphorylation of EGFR and Akt pathways. Increased expression of CDH5 is related to increased angiogenesis in lung cancer cells. Furthermore, both exon 19 deletion and exon 21 missense mutant EFGR genes promote migration and invasion of lung cancer cells. Our study may help to elucidate the association of EGFR mutations and CDH5 with angiogenesis and metastasis in lung cancer cells.

## Abbreviations

NSCLC: non-small cell lung cancer
EGFR: epidermal growth factor receptor
CDH5: cadherin-5
RT-PCR: real time polymerase chain reaction
TGF-α: transforming growth factor-α
VEGF: vascular growth factor
IL-6: interlukin-6
IL-8: interlukin-8
